# *In-vivo* magnetic resonance spectroscopy of lactate as a non-invasive biomarker of dichloroacetate activity in cancer and non-cancer central nervous system disorders

**DOI:** 10.3389/fonc.2023.1077461

**Published:** 2023-03-17

**Authors:** David O. Kamson, Viveka Chinnasamy, Stuart A. Grossman, Chetan Bettegowda, Peter B. Barker, Peter W. Stacpoole, Georg Oeltzschner

**Affiliations:** ^1^ The Sidney Kimmel Comprehensive Cancer Center, Johns Hopkins University, Baltimore, MD, United States; ^2^ Department of Neurology, Johns Hopkins University, Baltimore, MD, United States; ^3^ Department of Neurosurgery, Johns Hopkins University, Baltimore, MD, United States; ^4^ Russell H. Morgan Department of Radiology and Radiological Science, The Johns Hopkins University School of Medicine, Baltimore, MD, United States; ^5^ F. M. Kirby Research Center for Functional Brain Imaging, Kennedy Krieger Institute, Baltimore, MD, United States; ^6^ Departments of Medicine and Biochemistry and Molecular Biology, College of Medicine, University of Florida, Gainesville, FL, United States

**Keywords:** dichloroacetate, magnetic resonance spectroscopy, Warburg effect, lactate, glutamate, cancer, glioblastoma, glioma

## Abstract

The adverse effects of lactic acidosis in the cancer microenvironment have been increasingly recognized. Dichloroacetate (DCA) is an orally bioavailable, blood brain barrier penetrable drug that has been extensively studied in the treatment of mitochondrial neurologic conditions to reduce lactate production. Due to its effect reversing aerobic glycolysis (i.e., Warburg-effect) and thus lactic acidosis, DCA became a drug of interest in cancer as well. Magnetic resonance spectroscopy (MRS) is a well-established, non-invasive technique that allows detection of prominent metabolic changes, such as shifts in lactate or glutamate levels. Thus, MRS is a potential radiographic biomarker to allow spatial and temporal mapping of DCA treatment. In this systematic literature review, we gathered the available evidence on the use of various MRS techniques to track metabolic changes after DCA administration in neurologic and oncologic disorders. We included *in vitro*, animal, and human studies. Evidence confirms that DCA has substantial effects on lactate and glutamate levels in neurologic and oncologic disease, which are detectable by both experimental and routine clinical MRS approaches. Data from mitochondrial diseases show slower lactate changes in the central nervous system (CNS) that correlate better with clinical function compared to blood. This difference is most striking in focal impairments of lactate metabolism suggesting that MRS might provide data not captured by solely monitoring blood. In summary, our findings corroborate the feasibility of MRS as a pharmacokinetic/pharmacodynamic biomarker of DCA delivery in the CNS, that is ready to be integrated into currently ongoing and future human clinical trials using DCA.

## Introduction

The precision oncology era catalyzed enormous increases in tissue-based predictive biomarkers. Yet, the lagging development of non-invasive biomarkers remains a challenge in cancers where tissue access is limited, such as in the central nervous system (CNS). Advanced imaging may help overcome these issues by providing pharmacokinetic and/or pharmacodynamic information. Additionally, spatial mapping of drug delivery may allow customization of localized therapies to treat regions with inadequate drug accumulation and enhance our understanding of how drug resistance emerges in space and time.

**Figure 1:**
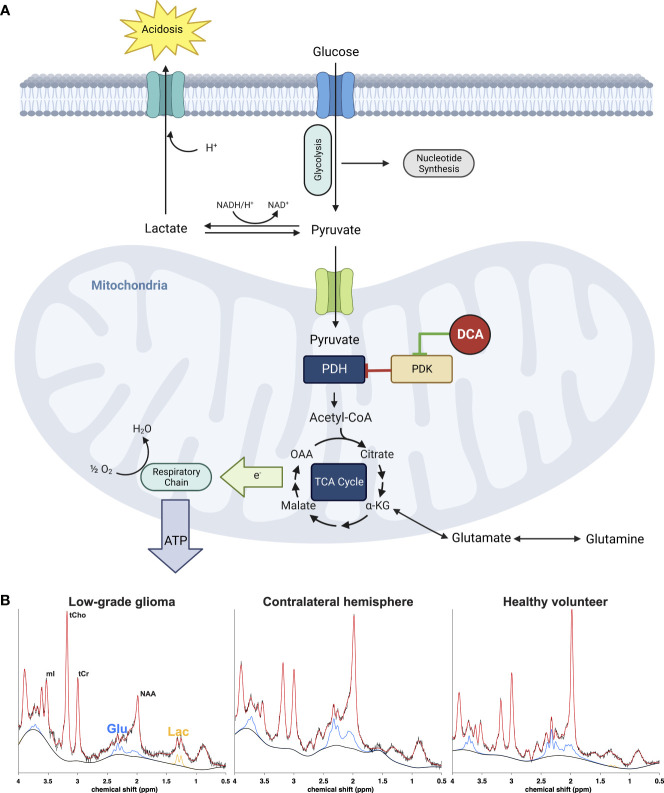
**(A)** Effect of DCA on metabolic pathways of cancer cells. Inhibition of the pyruvate dehydrogenase (PDH) complex by pyruvate dehydrogenase kinase (PDK) is responsible for the aerobic glycolysis observed in cancer, also known as the Warburg phenomenon. Accumulation of cytosolic pyruvate is converted to lactate which contributes to the acidification of the extracellular environment or utilized for anabolic processes necessary for cancer cell growth and proliferation. DCA inhibits PDK, activating the PDH complex and thus forcing the cancer cell to allow pyruvate to enter the tricarboxylic acid cycle and eventually terminal oxidation to generate ATP and restore the apoptotic machinery of the mitochondrion. The metabolic consequence is a rapid reduction of extra and intracellular lactate as well as increase of glutamate as the cell no longer has to rely on the latter as an alternative carbon source for the TCA cycle. **(B)** Representative 1H-MRS spectra from a low-grade glioma (left) normal appearing brain from the same patient (middle) and healthy normal volunteer (right). The signals from glutamate (Glu, blue) and lactate (Lac, orange) can be monitored after DCA administration to assess efficacy of drug delivery.

Uncovering the molecular mechanisms of the Warburg effect (aerobic glycolysis) – the commonly observed shift cancer cells undergo towards glycolytic ATP production – reignited research in the early 2000s (1) into the pharmacological metabolic reprogramming of cancer cell metabolism as a potential therapeutic approach. A salient feature of Warburg metabolism in tumors is the increased expression of one or more of four isoforms of pyruvate dehydrogenase kinase (PDK 1-4) that reversibly phosphorylate and thus inhibit the mitochondrial pyruvate dehydrogenase complex (PDC), which catalyzes the rate-limiting oxidation of glycolytically-derived pyruvate to acetyl coenzyme A (**Figure 1A**). Because cytoplasmic pyruvate and lactate are in equilibrium, inhibition of PDC also results in intracellular accumulation of lactate. Upregulation of PDK and consequent elevation of lactate levels play a major role in the glycolytic reprogramming seen in the cancer microenvironment (2) that is associated with decreased apoptotic function (3), invasive phenotypes (4, 5), decreased antitumor immunity (6) and shorter survival in animal models (7) and patients with various cancers (8, 9).

Here, we review evidence from *in vitro*, animal, and human studies on the use of magnetic resonance spectroscopy (MRS) to monitor the effects of dichloroacetate in neurologic and oncologic disorders. Additionally, we emphasize how MRS may be applied to enhance neurooncologic investigations and treatment paradigms.

### Dichloroacetate as an anticancer drug

Sodium dichloroacetate is a lipophilic small molecule (151Da) with high oral bioavailability that can readily pass the blood-brain barrier (10). Decades of research have established DCA to be a safe, well-tolerated investigational drug that is now in a phase 3 trial in children with congenital PDC deficiency (NCT02616484) and in a phase 2A trial in adults with recurrent glioblastoma (NCT05120284). As a pan-PDK inhibitor, DCA reverses Warburg metabolism and stimulates oxidative phosphorylation (OXPHOS) and apoptosis in cancer cells (**Figure 1A**) (11). Through its action on the PDC, DCA is also a potent lactate-lowering agent under many physiological and pathological conditions (12). The fall in tissue or circulating lactate concentration has been used as a biomarker of the PDC’s activation by DCA, including in tumors, in which DCA may also increase glutamate levels. Such metabolic changes could be exploited as a non-invasive, predictive monitor of both DCA’s delivery to tissues and its potential therapeutic efficacy. MRS is a well-established, widely available, non-invasive, radiation-free, and relatively low-cost method that allows spatial and longitudinal mapping of lactate, glutamate and other metabolites in tissue and thus could be a highly practicable method to monitor DCA dynamics in the clinic.

### Magnetic resonance spectroscopy

MRS applies the nuclear magnetic resonance effect to collect biochemical information from tissue. Many important biochemicals can be distinguished by characteristic peak patterns generated by chemically distinct nuclei in external magnetic fields. The nuclei most frequently targeted with *in-vivo* MRS are ^31^P (phosphorus) and ^1^H (proton), which give access to important metabolites involved in glycolytic energy generation, e.g., lactate, glutamate, and phosphocreatine (**Figure 1B**).

Exogenous administration of signal-enhancing isotope-labeled substrates (e.g., [^13^C]) enables the study of temporal dynamics of metabolism *in vivo via* tracking downstream metabolites based on their varying isotopic compositions (isotopologues) or configurations (isotopomers). The most commonly used substrate is hyperpolarized ([1-^13^C]pyruvate), that is converted to [^13^C]lactate or [^13^C]bicarbonate, where the lactate-bicarbonate ratio is interpreted as a marker for the imbalance between aerobic glycolysis and oxidative phosphorylation in cancer (13, 14). Another, but less commonly used substrate is hyperpolarized [2-^13^C]pyruvate, whose carbon label is incorporated into acetyl-CoA allowing a more distal view into the TCA cycle, or fatty acid synthesis that is upregulated in gliomas (15).

MRS is inherently quantitative. Signal amplitudes are directly proportional to molecular concentrations, although instrument and operator characteristics introduce (often unknown) scaling factors. Concentration estimates obtained with MRS are therefore reported relative to an internal reference standard, e.g., the total creatine or tissue water signal.

MRS has an *in vivo* detection threshold on the order of magnitude of 1 mM. *In vivo* measurements usually require a delicate trade-off between sufficient signal-to-noise, tolerable acquisition times, and spatial specificity. Single-voxel measurements may achieve robust estimation of lactate from an 8-mL cuboid in 5-10 mins; 2D/3D coverage of larger regions (‘spectroscopic imaging’, MRSI) at a spatial resolution of ~1 cm can require up to 20 to 30 minutes. The brain is an optimal organ for MRS because of its less intense lipid nuisance signals, less physiological motion, and the ability to achieve superior magnetic field homogeneity, compared to other body parts.

Proton *in vivo* MRS can be routinely performed on clinical MRI scanners while heteronuclear (i.e. non-proton) scans and hyper-polarization require additional equipment and expenses.

## Methods

We conducted a comprehensive literature search of PubMed and Google Scholar, and reference lists of identified studies on April 25, 2022, to identify research papers exploring the metabolic effect of DCA in mitochondrial, neurological, and cancerous conditions. Search criteria included various iterations of “MRS”, “dichloroacetate”, “cancer”, “glioma”, brain”. Specific search parameters are included in the supplement of this manuscript. Two authors (DK, GO) independently screened and selected titles and abstracts to classify MRS studies on the effects of DCA on lactate levels in the brain and blood.

Studies were included based on the following criteria: (a) interventional study design with DCA administration; (b) study of normal brain, mitochondrial disease, ischemia, stroke, TBI, and/or cancer; (c) utilization of MRS; (d) reported metabolite levels in the specimen/blood and/or brain. Duplicates were excluded.

Three reviewers (GO, DK, VC) then independently performed full-text screening and data extraction. Study characteristics for extraction included description of study population, condition examined, study design, number of participants, MRS method used, methodology for DCA administration, blood lactate levels pre-/post-DCA treatment, and brain lactate levels both pre-DCA and post-DCA. Disagreements were resolved through discussion among the reviewers. Studies are summarized in **Table 1**.

**Table 1:** Metabolic Effects of DCA in Non-Cancerous and Cancerous Conditions.

#	Condition of Interest, *Study Type*, (Reference)	Population	MRS technique	DCA Protocol	Summary
1	Normal – Whole Body, *animal model* (15)	Male Sprague-Dawley rats	Hyperpolarized [1-^13^C]Pyruvate [2-^13^C]Pyruvate	150 mg/kg DCA intraperitoneally after baseline imaging, and the second dose 5 min before follow-up (1.5 hours total duration)	50mg/kg HED DCA total administered IP and IV, with increased bicarbonate (181%), glutamate (74%) and acetoacetate (504%) levels but no change in lactate(+alanine) levels relative to pyruvate.
2	Normal – Brain, *animal model* (14)	5 healthy male Winstar rats	Hyperpolarized [2-^13^C]Pyruvate	150 mg/kg DCA given as a 0.5 mL bolus and then infusion at 0.1 mL/3 min.	Proton MRS study measuring mitochondrial metabolism in normal brain. A 40% increase of hyperpolarized glutamate was seen in the brain.
3	Cerebral Ischemia, *animal model* (16)	Male Sprague-Dawley rats	^31^P MRS and ^1^H NMR	100 mg/kg DCA infused over 10 min either 30 min before or 60 min after the start of ischemia	Postischemic administration of DCA started reducing lactate and pH within 30-40 minutes Maximum effect relative to controls was achieved 1 hour post reperfusion. Lactate levels were lower in the DCA treated group. MRS consistently detected ischemic and postischemic lactate even at the lowest measured levels (~1.5-2mM range). Glutamate levels were higher in the DCA group; however, the published MR spectra did not seem to indicate changes in Glu peak.
4	Cerebral Ischemia, *animal model* (17)	newborn swine and 1-month old swine.	^31^P MRS and ^1^H NMR	40 mg/kg DCA given following ischemia induction and 160 mg/kg given 5-7 min after deflation of the cervical cuff.	Effect of DCA on postischemic cerebral lactate clearance was studied. Nonsignificant trends toward faster lactate normalization in 1-mo old swine compared to controls on the ^1^H MRS analysis. Reduction in lactate levels was seen in the newborn swine relative to the 1-mo.
4	Traumatic Brain Injury, *animal model* (18)	5 Male Sprague-Dawley rats and 1 Wistar rat	Hyperpolarized [1-^13^C]Pyruvate	200 mg/kg DCA for 45 minutes. Repeated experiments on days 2, 7, and 28 on surviving rats	Head trauma was induced in rat, followed by DCA administration. 45min after DCI infusion, increased bicarbonate signal but no change in lactate signal on Hyperpolarized pyruvate MRI. Lactate/bicarbonate ratio changes were significant on the second day of DCA treatment.
5	Primary Mitochondrial Disorders (e.g., Leigh syndrome), *human* (19)	Human patients with a clinically diagnosed mitochondrial disorder	^31^P MRS of muscle and ^1^H NMR of brain	25 mg/kg DCA administered in capsules 2x per day for 1 week. 3-month intermediate period before the 2nd treatment course	In human mitochondrial disease DCA treatment reduced blood lactate by ~1mM on average and Lac/Cr ratio by 42%. Metabolic response on MRS was both seen in white matter and basal ganglia.
6	Ischemic Stroke, *human* (20)	Adult patients with acute ischemic stroke	^1^H NMR	Random assignment to four doses of DCA (60, 100, 150, and 200 mg/kg) for 15-30 minutes	1. In human acute stroke, 1–5-day post presentation, DCA doses 150-200mg/kg seemed to reduce Lac/NAA ratio by 19-25% however at a sample size was very limited (19 total, 8 patients in the high dose DCA groups) and statistical significance was not reached. 2. The study applied DCA in subacute ischemia, was agnostic of reperfusion status and obtained second MR spectrum about 45min after DCA infusion started.
7	Leigh Syndrome, *human* (21)	Case study on a 13-month-old patient with Leigh Syndrome	MRS (type not specified)	DCA was orally administered at 75 mg/kg per day for several months (not specified)	Lactic acid levels in CSF significantly lowered after DCA was started. The authors found benefit in monitoring both blood lactate and DCA levels, especially as the latter tends to accumulate in tissue over time.
8	Complex I Deficiency, *human* (22)	Case study on a 13-year-old patient with complex I deficiency	^1^H NMR and MRI	100 mg/kg DCA	Combination of DCA and thiamine reduced lactate concentration in blood in the CNS on MRS without objective clinical benefit. Peripheral neuropathy attributed to DCA toxicity had developed MRS of the brain normalized.
9	MELAS, *human* (23)	Case study on a 40-year-old patient with MELAS	^1^H NMR	50mg/kg DCA per day for 13 days, then 25 mg/kg per day for 6 days	MRS could initially detect elevated brain lactate in stroke-like region. Response to DCA therapy was seen as decreasing lesional lactate on MRS. Concurrent clinical response was seen. In a brain region with previously normal lactate levels, a newly developed symptomatic lesion that had double the Lactate-to-creatine ratio
10	Leigh Syndrome, *human* (24)	Case study on a 9-mo patient with Leigh Syndrome	^1^H NMR	30 mg/kg per day for an unspecified number of days	Blood/CSF lactate rapidly decreased in the first 6 weeks of DCA treatment, brain lactate on MRS remained elevated, until the next measurement in 6 months when all 3 measurements normalized. The findings suggest differences in temporal dynamics of lactate levels in blood, CSF, and brain.
11	Leigh Syndrome, *human* (25)	Case study on an infantile patient with Leigh Syndrome	^1^H NMR	DCA was given orally at 50 mg/kg 3 times every 12 hours followed by 50 mg/kg/day for an unspecified number of days	DCA lowered lactate and pyruvate levels in blood and CSF, though patient developed cerebral atrophy over time. Lactate concentrations increased the most in the basal ganglia and brainstem.
13	Breast Cancer, *in vitro* (26)	Breast cancer cell lines (TS/A, 4T1, TUBO) and non-tumor cell lines (J774)	^1^H NMR using MRI CEST	treated with DCA (1, 5 and 10mM) for 24h	1. DCA reduced cell viability in a dose-dependent fashion in cancer cell lines both under normoxic and hypoxic circumstances but not in non-cancer cell lines. 2. DCA reduced lactate levels and extracellular pH consequently, and these changes were detectable on pH weighted CEST MRI but not on MRS.
14	Colorectal Cancer, *in vitro* (27)	HT29 colorectal cancer cells in female NCr nude mice	[1-^13^C]Pyruvate Hyperpolarized	Treated on days 1 and 2 with 200 mg/kg DCA or saline p.o. and a final dose was given 1 h before Day 3 measurement post-treatment	Study of colorectal cancer cell lines increased ROS, autophagy and mTOR inhibition after DCA treatment. In xenograft models, DCA (HED ~30mg/kg) elicited tumoristatic effect after 3 days, with pyruvate to lactate conversion rate reduced to 50% on hyperpolarized MRI
15	Prostate Cancer, *in vitro* (27)	PC3 and HT29 prostate carcinoma cells	[1-^13^C]Pyruvate Hyperpolarized	80 mM and 100 mM respectively for 24 hours	
16	Colorectal Cancer, *in vitro* (27)	HT29, HCT116 WT, and HCT116 Bax-ko human colorectal adenocarcinoma cells	[1-^13^C]Pyruvate Hyperpolarized	100 mM, 75 mM, and 75 mM respectively for 24 hours	
17	Prostate Cancer, *in vitro* (28)	LNCaP and LNCaP-LN3 human prostate cancer cell lines	^1^H NMR	50 mM DCA was used to treat cultures for 24 hours	Prostate cancer cell lines deemed to have low vs. high metastatic potential, DCA induced: Insignificant reductions in lactate in the low metastatic and marked lactate reduction in the high metastatic cell line
18	Cervix Squamous Cell Carcinoma, *in vitro* and a*nimal model* (29)	Female mice bearing MDA-MB-231 human breast cancer xenografts and NMR	^17^O MRS and hyperpolarized [1-^13^C]pyruvate	200 mg/kg administered after baseline measurements and again 24 hours after first dose	In a glycolytic breast cancer and oxidative cervical cancer in vivo murine model, 2 days of treatment with 200mg/kg/d (15mg/kg HED) tumor models, did not majorly affect oxygen consumption, glucose utilization, whereas pyruvate to lactate conversion was paradoxically only reduced in the oxidative cell line.
19	Breast Cancer, *in vitro* and a*nimal model* (29)	Female mice bearing SiHa human cervix squamous cell carcinoma xenografts and NMR	^17^O MRS and hyperpolarized [1-^13^C]pyruvate	200 mg/kg administered after baseline measurements and again 24 hours after first dose	
20	Pancreatic Cancer, *in vitro* (30)	9850 and 10158 PI3K driven pancreatic cancer cell lines	hyperpolarized [U-^13^C_6_]glucose	75mM DCA for 18 hours	Hyperpolarized pyruvate and 75 mM DCA for 18 h in a pancreatic cancer cell line. DCA significantly reduced glucose consumption in examined amino acids indicating massive inhibition of glycolytic flux.
21	Glioblastoma, *in vitro* (31)	U87 and NHA IDH1 mutant glioblastoma cells	Hyperpolarized 2-^13^C -pyruvate	10 mmol/L DCA for 24 hours	Hyperpolarized pyruvate used to study glucose and glutamine changes induced by DCA in an IDHwt versus an IDHmut glioma cell line. Pyruvate Dehydrogenase (PDH) had a 4-fold lower activity in the IDHmut cell line with concurrent overexpression of PDK mRNA. DCA reactivated PDH more than 700% in in in IDHmut, but only 41% in IDHwt cell lines with 2.4-5.9-fold increase in glutamate levels in IDHmut but only marginal increases in IDHwt. DCA reduced the colony forming effect and proliferation of IDH mutant cell lines.
22	C6 Glioma, *animal model* (13)	Healthy and C6 glioma implanted Wistar rat brains	Hyperpolarized [2-^13^C]Pyruvate	200 mg/kg of DCA administered as a 0.5-mL bolus followed remaining infusion of 0.1 mL/3 min	Hyperpolarized pyruvate MRS in a glioma xenograft model. Glutamate signal: 50% increased 1 hour and 86% at 2.5H post DCA injection. Lactate signal: decreased by ~30% 1 hour but returned to the preinjection levels 2.5 hours post injection in the normal appearing brain but was unchanged on an alternative aliasing scheme. Lactate/Glutamate ratios were about 30-40% higher in gliomas than in normal appearing brain and doubled after DCA in both glioma and normal brain.

### DCA and MRS in normal brain and non-oncological neurologic disorders

DCA is a drug of interest to modulate CNS metabolism and its derangements and thus has been extensively studied in the treatment of mitochondrial and ischemic neurologic conditions. Many preclinical and clinical studies also evaluated the potential role of MRS as a pharmacodynamic biomarker for DCA-induced metabolic changes. Such studies may serve as a blueprint for further development of MRS for DCA treatment monitoring in oncology.

Rat models studied with hyperpolarized pyruvate MRS have been particularly useful to establish the dynamics of DCA-related metabolic changes. In normal rats, whole-body measurements after DCA administration revealed a ~three-fold rise of bicarbonate and 70% increase of glutamate levels (15), and 40-50% rise of glutamate in normal rat brain after 1 hour, continuing further to 86% 2.5 hours after injection (13, 14). Brain lactate concurrently decreased by ~30% one hour after DCA injection but returned to pre-injection levels by 2.5 hours post-injection (13). A similar approach revealed a diffuse rise in bicarbonate and bicarbonate/lactate ratios after traumatic brain injury (TBI) in rats, although the changes appeared global, without significant differences between the injured or non-injured hemispheres in the acute (4 hours) or subacute (2 and 7 days) phases after injury (18).

In a rat model of cerebral ischemia, lactate levels as low as 1.5 mM were detectable with MRS, and lower lactate with higher pH were observed 30 minutes after reperfusion in the DCA-treated versus the DCA-naïve group. Preischemic DCA treatment only minimally lowered lactate during the occlusive phase, but pre- or post-occlusive DCA treatment induced marked lactate reduction that was most prominent in the first hour of reperfusion. Glutamate levels were higher in the DCA-treated group than in DCA-naïve postischemic animals (16). In another MRS study of cerebral ischemia comparing adolescent and adult mice, DCA use was associated with mostly nonsignificant trends towards faster lactate normalization after reperfusion in the adolescent mice (17).

Human DCA/MRS data is dominated by case reports of pediatric mitochondrial conditions treated with DCA. Leigh syndrome is characterized by elevated blood lactate levels and symmetric deep brain lesions secondary to global PDC or respiratory chain defects (32). Three case studies, each of an infant with Leigh syndrome, used MRS qualitatively (21) and quantitatively (24, 25) to supplement data from serum and CSF lactate level monitoring while on DCA. Before initiation of DCA, two of these patients had borderline high (~1 mM), and one markedly elevated serum lactate levels (5.5 mM), and all had elevated CSF lactate (3.0-8.0mM) with clearly detectable MRS lactate signal, including the normal appearing parenchyma. At the first MRS timepoint on DCA therapy (30-100 mg/kg/day), serum lactate levels decreased or plateaued in the broad normal range within a week, whereas CSF lactate levels had a much slower rate of reduction and took substantially longer to plateau. MRS lactate peaks became undetectable when CSF lactate levels reached ≤2.0 mM (as early as 10 weeks) (21, 24, 25). Lactate-to-creatine ratios did not follow CSF lactate levels, but correlated with clinical responses, suggesting that MRS might be a clinically relevant biomarker in DCA use (24, 25). In the case of a 13-year-old with mitochondrial complex I deficiency and lactic acidosis, a dose-dependent reduction of serum lactate and disappearance of MRS lactate signal was noted upon administration of DCA (22).

Unlike the disorders above, ictal appearance of focal stroke-like lesions predominates in MELAS which is caused by mitochondrial DNA alterations (33). Clinical symptoms are due to focal rather than global mitochondrial dysfunction in MELAS and thus more adequate to study the spatial dynamics of lactate homeostasis in lesional diseases such as brain tumors. Consistent with this notion is the case of a 40-year-old woman with MELAS, who presented with lactic acidosis (serum lactate 4.0 mM) and refractory partial seizures, presumably emanating from a left occipital lobe lesion with a lactate-to-creatine ratio of 2.5, as determined by MRS (23). One week of 50 mg/kg/day oral DCA led to rapid clinical improvement, reduction of serum lactate (to ~1.7 mM) and progressive long-term reduction of the MRS lactate-to-creatine ratio (~1.8 at one week, ~0.4 after 3 more weeks of DCA) in the occipital lesion, even when serum lactic acidosis (2.5 mM) re-emerged after DCA discontinuation. The patient subsequently acquired a new neurologic deficit localizable to the other occipital lobe, which showed a newly elevated lactate-to-creatine ratio of 1.3. In a case series of 11 patients with various congenital mitochondrial conditions (e.g., Leigh’s, MELAS, Kearns-Sayre, mitochondrial myopathy), 25 mg/kg/day DCA reduced blood lactate by ~1 mM on average and brain lactate-to-creatine ratios by, on average, 42% in white matter and basal ganglia, although with heterogeneous individual effect size (19).

A prospective placebo-controlled clinical trial studied the administration of various doses of DCA in stroke 1-5 days after presentation to the hospital (20). Nineteen patients had an MRS performed before and 45 minutes after DCA administration. Although a statistically significant lactate reduction was not observed when the whole cohort was considered, the lactate-to-NAA ratio was lowered by 19-25% in patients treated with higher doses of DCA (150-200 mg/kg, n=4). Of note, this study applied DCA in subacute ischemia and was agnostic of reperfusion status. As indicated by animal data (16), the degree of reperfusion significantly affects the lactate-reducing effect of DCA and may have confounded the findings of this human study (20).

In summary, human and animal evidence suggests that normal, non-cancer tissues (including the brain) respond to DCA treatment with a marked increase of glutamate and reduction of lactate. In primary mitochondrial disorders, especially in those with focal lesions, MRS estimates of brain parenchymal lactate levels follow a much slower rate of lactate reduction compared to changes measured in blood and may correlate better with clinical presentation than blood or CSF lactate. Lastly, DCA-associated metabolic changes appear to consistently follow a dose-dependent pattern across models and pathologies.

### The role of MRS in monitoring DCA response in cancer

#### Non-brain cancer models

There are much less data from MRS or other metabolic imaging methods regarding responses to DCA in cancer. An *in vitro* study of PIK3CA-driven prostate cancer cell lines assessed the impact of DCA treatment on carbon homeostasis using [^13^C]glucose MRS (30). It found DCA to increase carbon flux *via* the tricarboxylic acid (TCA) cycle, with concurrent inhibition of glycolytic flux and amino acid biosynthesis. DCA reduced tumor proliferation and viability in the studied cell lines at a relatively high half-maximal inhibitory concentration (IC50) of 75 mM. A combined *in vitro* and *in vivo* study of colorectal cancer found increased production of reactive oxygen species, increased autophagy and mTOR inhibition after DCA treatment in cell lines (27). When these were xenografted into rat, hyperpolarized MRSI measures of pyruvate-to-lactate conversion rate decreased by 50%, with concurrent tumoristatic effects at a DCA dose of 200 mg/kg/d (human equivalent dose [HED] ~30 mg/kg/d). Another study showed DCA to reduce vitality metrics and lactate levels in a dose-dependent fashion in breast cancer cell lines, but not in a non-neoplastic breast cell line (26). Here, DCA increased extracellular pH in cell cultures from 7.06 to 7.23, pH and lactate levels under normoxic conditions were strongly correlated (r=-0.98), and only slightly weaker under hypoxia (r=-0.93). DCA had minimal impact on cell-cycle in the cultured cells. DCA-treated mice had a similar rise in extracellular pH with statistically insignificant growth reduction and longer survival compared to DCA-naïve animals (26). In another murine study of glycolytic breast cancer and oxidative cervical cancer xenografts, 2 days of treatment with 200 mg/kg/day (15 mg/kg/d HED) did not impact oxygen and glucose utilization significantly. Moreover, there was no difference in double-stranded DNA breaks between the cell lines, and a pyruvate-to-lactate conversion reduction on MRS was only noted in the oxidative cell line (29).

#### Brain cancer models

There are a few preclinical studies that have evaluated the use of hyperpolarized MRS and DCA in U87 and C6 glioma models. In a study of hyperpolarized glucose and glutamine metabolism in glioma cell lines, hyperphosphorylated PDC showed a 4-fold lower activity in the IDH-mutant cell line with concurrent overexpression of PDK mRNA relative to IDH-wildtype (31). In IDH-wildtype cell lines, DCA induced a 41% increase in PDC activity and only marginal increases in glutamate levels. In contrast, DCA reactivated PDC by more than 700% and increased glutamate levels 2.4-5.9-fold in the IDH-mutant group and reduced the colony-forming effect and proliferation.

MRS of hyperpolarized pyruvate revealed reduced bicarbonate in both tumor and normal-appearing brain in an orthotopic C6 glioma animal model (34). Lactate levels normalized to total carbon did not significantly change in normal brain but decreased by 11% in the tumor (34). In the same model, lactate-to-glutamate ratios were about 30-40% higher in gliomas than in normal appearing brain at baseline. DCA administration led to a >50% reduction of these ratios in both glioma and normal brain (13).

Overall, these preclinical data from various cancer models suggest that DCA can increase extracellular pH both *in vitro* and *in vivo* and may shift glucose utilization from glycolysis to OXPHOS. However, the impact on tumor cell proliferation and viability varied markedly among cell lines. In gliomas, there appears to be a significant differentiating effect of higher lactate-to-glutamate ratios that respond to DCA with concurrent reductions of lactate and increases in glutamate levels within 1 hour of drug administration. Lastly, the impact of DCA on PDC activity was more than 17-fold higher in IDH-mutant glioma that also translated to higher tumoristatic effects *in vitro*. However, hyperpolarized MRS data has yet to be substantiated with conventional (non-hyperpolarized) clinical MRS in humans.

## Discussion

The evidence reviewed here shows a consistent and apparently dose-dependent effect of DCA on serum and tissue lactate and glutamate levels. Data derived from various neoplasms indicate that tumor sensitivity to these effects may strongly depend on tumor type and metabolic environment, including oxygenation status, but are substantial in gliomas. Experience from human primary mitochondrial diseases suggests that brain lactate levels estimated by MRS correlate with clinical responses to DCA therapy better than serum or even CSF lactate, especially in conditions where symptomatology is driven by focal lesions similarly to brain tumors. Therefore, it is unlikely that serum and/or CSF lactate measurements could serve as surrogate biomarkers of DCA pharmacodynamics in brain cancer, or possibly cancer located in other tissues. Although lactate MRS detection thresholds were not clearly evaluated in the above reports, MRS lactate signals were detectable in all animal or human studies when tissue or CSF lactate exceeded >2 mM, which is well below lactate concentrations measured in human gliomas (>5 mM) or even peritumoral brain (≥2 mM) (35). Another important finding is the marked increase in glutamate concentrations upon DCA exposure in both normal and tumor-bearing brain in animal models. The inverse correlation between lactate and glutamate may be explained by the role of the glutamate as an alternative carbon source for the TCA cycle when pyruvate carbon is depleted by lactate production in tumor tissue (36). These data suggest that, by stimulating pyruvate oxidation, DCA may inhibit glutamine oxidation by decreasing the availability of pyruvate for transamination, which, in turn, results in glutamate accumulation and a consequent inhibition of glutaminase activity, although this postulate remains to be tested definitively. Regardless, the temporal overlap 1 hour after DCA administration between lactate reduction and glutamate increase could be harnessed to assess adequate distribution of DCA in normal brain and the solid portion of gliomas. The utility of this approach was demonstrated in a human dose-finding trial of a dual mTOR/PIK3CA inhibitor, in which the glucose uptake-reducing effect measured on PET was used to inform dose selection (37). The present data could serve as an excellent framework to design prospective, tissue-validated studies of the role of MRS as a predictive biomarker in DCA therapy in cancers and neurologic conditions, such as stroke.

## Conclusion

The evidence suggests that DCA is a well-tolerated, orally bioavailable, brain-penetrable compound with remarkable effects on brain lactate and glutamate levels. Rapid DCA-induced reduction of lactate levels has been shown repeatedly in cancer and appears promising as lactic acidosis is associated with worse survival and treatment resistance in malignancies such as glioblastoma (38). The data also corroborate that lactate and glutamate changes are well within the detection range of MR spectroscopy. Consequently, monitoring these metabolites *via* MRS appears to be a feasible approach to confirm adequate DCA delivery and kinetics in the brain that is ready to be integrated into currently ongoing and future human clinical trials using DCA.

## Author contributions

DK, concept, writing, literature review, editing figure and table VC, literature review, table and figure generation, formatting, GO, concept, writing, literature review, editing figure and table PS, literature interpretation, writing, editing, SG: concept, literature interpretation, editing PB: concept, literature interpretation, editing CB: concept, literature interpretation, editing. All authors contributed to the article and approved the submitted version.
