# Supplementary material for: In-vivo magnetic resonance spectroscopy of lactate as a non-invasive biomarker of dichloroacetate activity in cancer and non-cancer central nervous system disorders
Source: Front Oncol. 2023 Mar 17;13:1077461. doi: 10.3389/fonc.2023.1077461 (PMC10063958; doi:10.3389/fonc.2023.1077461)

Supplementary Materials

Supplements 1:

The following search criteria were used: Google Scholar: ("MR spectroscop*" OR "magnetic resonance spectroscop*" OR "MRS" OR "MRSI") AND ("dichloroacetate") AND ("lactate" OR "lactic acid") AND ("brain" OR "CNS" OR "central nervous system" OR "cancer" OR "glio*" OR "astrocyt*" OR "carcinoma" OR "tumor" OR "GBM"); Pubmed: ("MR spectroscopy" OR "MR spectroscopic" OR "magnetic resonance spectroscopy" OR "magnetic resonance spectroscopic" OR "MRS" OR "MRS") AND ("dichloroacetate") AND ("brain" OR "CNS" OR "central nervous system" OR "cancer" OR "glio*" OR "astrocyt*" OR "tumor" OR "brain metastas*" OR "neoplas*" OR "GBM").

Supplemental Figure 1: PRISMA flow chart. A total of 175 studies were imported. Of these 4 duplicates were removed, and the remaining 171 studies were screened. Following title and abstract screening, 139 studies were found irrelevant according to inclusion criteria. 30 full-text studies were reviewed for eligibility, of which 5 were excluded for interventional and design inconsistencies with inclusion criteria. Finally, 25 studies were included.

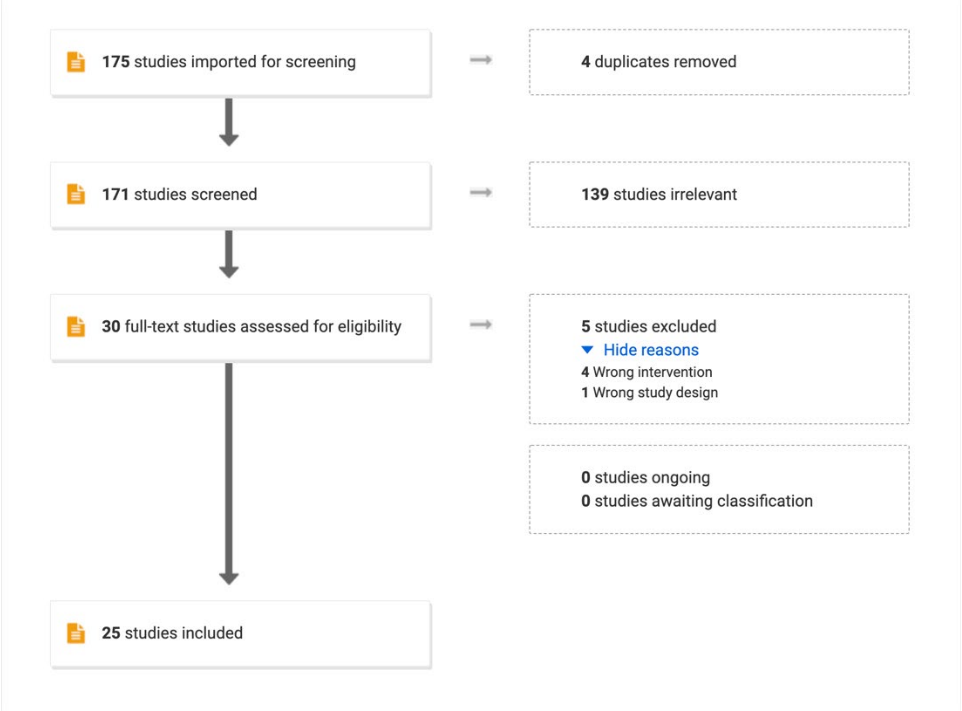

Supplement: Supplementary file 1 [file DataSheet_1.pdf]
